# Myelodysplastic Syndromes and Metabolism

**DOI:** 10.3390/ijms222011250

**Published:** 2021-10-19

**Authors:** Ekaterina Balaian, Manja Wobus, Martin Bornhäuser, Triantafyllos Chavakis, Katja Sockel

**Affiliations:** 1 Medical Department I, University Hospital Carl Gustav Carus, Technische Universität Dresden, 01307 Dresden, Germany; manja.wobus@uniklinikum-dresden.de (M.W.); Martin.Bornhaeuser@uniklinikum-dresden.de (M.B.); 2 German Cancer Consortium (DKTK), Partner Site Dresden and German Cancer Research Center (DKFZ), 69120 Heidelberg, Germany; 3 National Center for Tumor Diseases, Partner Site Dresden and German Cancer Research Center (DKFZ), 69120 Heidelberg, Germany; Triantafyllos.Chavakis@uniklinikum-dresden.de; 4 Institute for Clinical Chemistry and Laboratory Medicine, University Hospital Carl Gustav Carus Dresden, 01307 Dresden, Germany

**Keywords:** myelodysplastic syndromes, metabolism, inflammation

## Abstract

Myelodysplastic syndromes (MDS) are acquired clonal stem cell disorders exhibiting ineffective hematopoiesis, dysplastic cell morphology in the bone marrow, and peripheral cytopenia at early stages; while advanced stages carry a high risk for transformation into acute myeloid leukemia (AML). Genetic alterations are integral to the pathogenesis of MDS. However, it remains unclear how these genetic changes in hematopoietic stem and progenitor cells (HSPCs) occur, and how they confer an expansion advantage to the clones carrying them. Recently, inflammatory processes and changes in cellular metabolism of HSPCs and the surrounding bone marrow microenvironment have been associated with an age-related dysfunction of HSPCs and the emergence of genetic aberrations related to clonal hematopoiesis of indeterminate potential (CHIP). The present review highlights the involvement of metabolic and inflammatory pathways in the regulation of HSPC and niche cell function in MDS in comparison to healthy state and discusses how such pathways may be amenable to therapeutic interventions.

## 1. Introduction

Myelodysplastic syndromes (MDS) are a heterogeneous group of acquired clonal stem cell disorders that have an increased prevalence in the aging population with an incidence of 40–50/100,000 inhabitants in patients aged ≥70 years [1]. Patients are primarily affected by cytopenia-related symptoms, such as fatigue due to anemia and bleeding due to thrombocytopenia or neutropenia-related infections. In approximately 30% of MDS cases, the disease progresses to acute myeloid leukemia (AML) [2]. Currently, only limited treatment options are available, and only five drugs are approved in Europe (erythropoietin, luspatercept, the iron chelator deferasirox, the immunomodulatory drug lenalidomide, and the hypomethylating agent azacytidine). The only curative therapy for MDS is allogeneic hematopoietic stem cell (HSC) transplantation [3]. Clonal hematopoiesis of indeterminate potential (CHIP) is a condition related to MDS. It is defined as the age-related accumulation of somatic mutations in HSPCs that are usually linked to myeloid malignancies, such as MDS, yet these patients do not have symptoms or cytopenia; however, CHIP enhances the risk of myeloid malignancies [4,5].

During the last years, alterations in cellular metabolism have been implicated both in cancer development and in the maintenance of a tumor-promoting microenvironment in several malignancies [6,7,8]. In this context, mitochondria are the central hub of metabolic cell regulation, hosting the tricarboxylic acid cycle (TCA) and oxidative phosphorylation (OXPHOS) [9]. Indeed, the cellular metabolism of HSPCs is integral to the regulation of fate decisions, including self-renewal versus cell differentiation [10], which are tightly balanced under normal conditions. Similarly, cell metabolism may orchestrate the hematopoietic response to stress conditions (such as infection or inflammation) [10]. This inflammatory adaptation of HSPCs is also relevant in non-hematologic diseases, e.g., chronic inflammatory pathologies, and may potentially link inflammatory comorbidities to hematopoietic malignancies [11,12,13].

HSC maintenance and function are supported by the hematopoietic niche [14], which contains extracellular matrix and cellular components, including mesenchymal stromal cells (MSCs), osteoprogenitors, endothelial cells, macrophages, adipocytes, and osteoclasts [14,15,16]; these components regulate HSPC function via direct cellular interactions or paracrine secretion of cytokines and other factors. The niche is also able to adapt to inflammatory signaling, such as through the upregulation of osteoclastogenesis, which may result in increased bone resorption, and the promotion of cells of the monocytic lineage to produce higher levels of pro-inflammatory cytokines [11].

Increasing evidence suggests that inflammatory and metabolic dysregulation of HSPCs and niche cells may contribute to MDS pathogenesis. In this review, we will therefore discuss recent findings regarding metabolic changes and their association with inflammatory processes in the context of MDS as opposed to the healthy state. We also provide possible implications for potential therapeutic targets.

## 2. Metabolic Changes in the HSPC Pool

HSCs are at the top of the hierarchical system of hematopoiesis and are characterized by their ability to proliferate without lineage commitment (termed self-renewal) and to differentiate into different blood cell types (pluripotency) [17]. HSCs can thereby replenish the hematopoietic system with mature differentiated blood cells throughout life, while the quiescent HSC pool is also maintained throughout life.

In response to different demands of the organism (such as infection, blood loss, acute inflammatory conditions, and other stress conditions), HSCs typically exit their quiescence and undergo proliferation and differentiation; this process is termed demand, stress, or emergency hematopoiesis [18]. The different HSC and HSPC functional states are associated with distinct metabolic demands and metabolic states [19]. The metabolic plasticity of HSPCs, as illustrated by the switch between glycolysis and mitochondrial OXPHOS, helps them cope with these demand-driven needs [19]. Notably, cells can adapt the quantity and activity of their mitochondria in response to their needs, which are also defined by environmental cues [20]. In the steady state, HSCs are *quiescent*, with reduced cellular turnover and low energy demands. Energy is primarily derived from glycolysis, whereas mitochondrial activity is relatively low [19]. In addition, the suppression of mitochondrial respiration further promotes the quiescent state along with self-renewal and a low susceptibility to cytotoxic and genotoxic stress [21]. In contrast, HSC proliferation and differentiation require a high-energy input; this is ensured by a metabolic shift from a predominantly glycolysis-based metabolism to mitochondrial metabolism, which results in higher energy generation. To this end, the adaptation of mitochondrial biogenesis, which is associated with enhanced NADH levels, the upregulation of TCA cycle enzymes, and the downregulation of glycolytic enzymes are required [22]. These adaptations result in an increased oxygen consumption rate and lower lactate levels [23]. In contrast, mitochondrial respiration is impaired by a deficiency of mitochondrial subunits, e.g., the mitochondrial complex III subunit Rieske iron-sulfur protein (RISP) [24] or mitochondrial enzymes, such as protein tyrosine phosphatase mitochondrial 1 (PTPMT1) [25]; the deficiency of the aforementioned factors inhibits HSC differentiation.

In addition to mitochondrial respiration, other metabolic pathways are involved in HSC differentiation and proliferation. When HSCs leave the quiescence state, they undergo symmetric or asymmetric division. While symmetric division results in two identical daughter cells with the same fate, asymmetric division produces one daughter cell that has the potential to differentiate and another daughter cell that still exhibits stem cell properties, e.g., self-renewal [26]. Inhibition of fatty acid oxidation (FAO) leads to an inability of HSCs to undergo asymmetric division [27]. Interestingly, specific metabolic molecules contribute to HSC lineage commitment. Some examples are molecules involved in glutamine transport and metabolism, which are essential prerequisites for erythroid differentiation, as short hairpin RNA (shRNA)-mediated knockdown of the *ASCT2* gene, which encodes a glutamine transporter, leads to myelomonocytic differentiation [28]. Another example is the accumulation of cholesterol in the cell membrane resulting from deficiency in cholesterol export systems, which enhances HSPC proliferation and myeloid differentiation by increased cell-surface expression of the common beta-chain of the receptor for IL-3 and GM-CSF [29].

### Metabolic Changes and Their Association with Genetic and Epigenetic Alterations in MDS

Clonal changes in the HSC compartment are a key event in the development of myeloid malignancies, as somatic mutations have been identified in up to 90% of MDS patients [30]. No MDS-defining mutation has been reported, but rather a subset of recurrently mutated genes. Nevertheless, the way in which mutations in different genes, encoding unrelated proteins may lead to similar clinical features, is debatable. One possible explanation may be the convergence of various genetic events in cases of similar metabolic alterations. As such, the aberrant hypoxia-independent expression of hypoxia inducible factor 1α (HIF1α) has been described to occur in at least five common MDS-related mutations (involving the genes *Dnmt3a*, *Tet2*, *Asxl1*, *Runx1*, and *Mll1*) [31]. This transcription factor is essential for the hypoxic response and HSC regulation and correlates with poor overall survival and disease progression in patients with MDS [32]. In genetically modified mice with an MDS phenotype, the metabolic reprogramming of HSCs involves an increase in extramitochondrial glucose catabolism despite adequate oxygen availability (aerobic glycolysis), which is known as the Warburg effect, a common metabolic pathway in cancer cells. This results in the activation of HIF1α through different intermediate metabolites of the TCA cycle, while mitochondrial biogenesis is suppressed [33], thus coupling genetic events to metabolic changes [31]. Notably, not only has the direct effect of HIF1α on HSCs been described, but indirect effects of HIF1α may occur through niche cell signaling (see Section 3, [34]).

**Figure 1:**
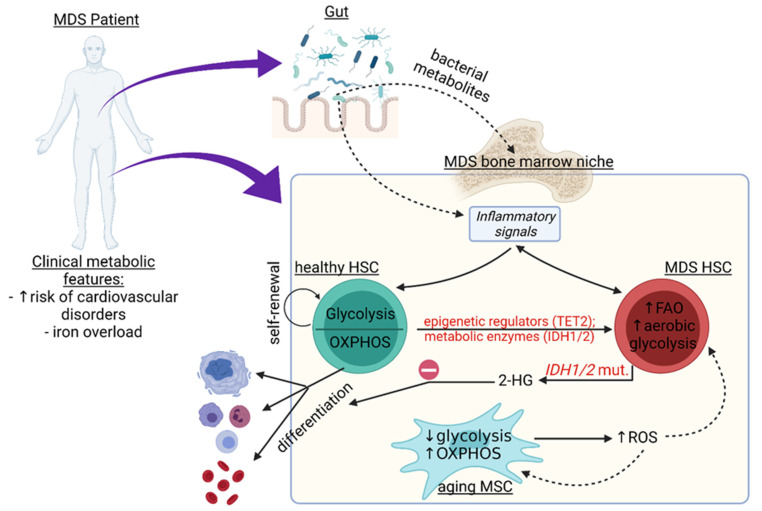
Metabolic changes in myelodysplastic syndromes (MDS). Patients with MDS clinically demonstrate the propensity to develop cardio-metabolic disturbances, such as cardiovascular disorders and iron overload. Various cellular and molecular mechanisms are responsible for the metabolic changes in these patients. Whereas the healthy hematopoietic stem cells (HSC) preferentially engage in glycolysis for their self-renewal, differentiation to mature blood cells is linked with oxidative phosphorylation (OXPHOS). MDS HSC increase the level of aerobic glycolysis and fatty acid oxidation (FAO). At least in part, epigenetic regulators, such as TET2, and metabolic enzymes, such as IDH1/2, regulate the shift in metabolic pathways; for example, the oncometabolite 2-hydroxyglutarate (2-HG) accumulates in IDH1/2 mutated cells. Regulation of such metabolic pathways may be involved in the ineffective erythropoiesis in MDS. Furthermore, the deterioration of MSC function in aging patients, accompanied by decreased glycolysis and increased OXPHOS, is associated with increased production of ROS by MSC, which are, in turn, capable of modifying proteins, lipids, and DNA in both MSC and HSC, leading to further expansion of the malignant clone. A possible role of bacterial metabolites from the gut microbiome in the maintenance of chronic inflammation and related metabolic changes in the bone marrow niche is conceivable.

Somatic mutations in genes encoding epigenetic regulators represent some of the most frequent mutations observed in MDS [30]. Epigenetic changes result in post-translational modification of DNA and histones. One of the most important epigenetic alterations in myelodysplastic syndromes is modification of DNA methylation, which can be targeted by hypomethylating agents, such as azacytidine [35]. Epigenetic modifications are regulated by metabolites, such as acetyl-coenzyme A (CoA), S-adenosylmethionine, α-ketoglutarate (α-KG), 2-hydroxyglutarate (2-HG), and butyrate, which act as substrates, cofactors, or antagonists in this context [36]. For example, excessive glycolysis coupled with defective OXPHOS and increased reductive carboxylation of glutamine in MDS is associated with elevated levels of the oncometabolite 2-HG [37] (Figure 1). This effect is particularly prominent in *IDH1*-mutated MDS and AML, where the defective IDH1 enzyme results in a very high accumulation of 2-HG rather than α-KG [38]. Recently, published data in mice have shown that 2-HG inhibits oxoglutarate dehydrogenase activity and reduces succinyl-CoA production, which leads to attenuation of heme biosynthesis and ineffective erythropoiesis [39].

The interactions between metabolism and epigenetic regulators are supposed to be bidirectional, and epigenetic modifications then drive metabolic changes and thus disease progression. Although data on metabolism in MDS are scarce, a recent publication described two different metabolomic profiles that can be used to differentiate myeloid cells of untreated MDS patients depending on the blast cell count, which indicates metabolic plasticity during disease evolution [40]. Although the Warburg effect has been detected in groups with <5% and >5% blasts, the metabolic outcomes of these groups were substantially different. In the group with a lower blast count, the accumulation of glycolytic metabolites was detected, whereas the group with a higher blast count demonstrated improved functioning of the electron transport chain, thus compensating for Warburg effect disruption [40]. This resembles the former conclusion that leukemic stem cells engage preferentially in oxidative phosphorylation, while healthy quiescent HSCs depend on glycolysis for energy production [41]. Altered lipid metabolism appears to be a prominent feature of the MDS phenotype, as indicated by extreme upregulation of phospholipids in the high blast count group, thereby providing a possible link to the risk of disease progression toward AML [40]. The breakdown of lipids, termed fatty acid β-oxidation (FAO), is increased in myeloid malignancies associated with enhanced autophagy, which, in turn, also supports OXPHOS specifically in malignant cells but not in normal cells; this leads to proliferation and growth of malignant clones [42]. Thus, pharmacological modulation of FAO, e.g., with the reversible FAO inhibitor malonyl-CoA, is considered a potential therapeutic intervention to modulate malignant cell fate and, possibly, to influence normal hematopoiesis [43].

## 3. Metabolic Pathways in Non-Hematopoietic Cells of the MDS Niche and Beyond

The hematopoietic niche is a key player that supports and regulates hematopoiesis by producing various cytokines and chemokines, secreting extracellular matrix components, and maintaining direct cell–cell interactions [14]. MSCs are one of the most important cellular players in the hematopoietic niche, as they have the potential to differentiate into cells of the adipogenic, osteogenic, or chondrogenic lineage [44]. In vivo, MSCs use glycolysis as the primary metabolic pathway and sustain “young” mitochondria through intensive autophagy and mitophagy [45]. One explanation for the utilization of glycolysis despite its reduced ATP production is the benefit of the protective effect of antioxidants from the pentose phosphate pathway [46]. MSCs may alter their metabolism in aging or disease. As such, MSCs derived from obese patients contain more defective mitochondria and exhibit reduced levels of glycolysis [47]. Similarly, MSCs of elderly individuals, especially those with atherosclerosis, favor OXPHOS, which leads to the accumulation of reactive oxygen species (ROS) [48,49]. Preference for OXPHOS is also observed in cells of mesenchymal origin after treatment with the hormone erythropoietin [50,51,52], which is elevated in a portion of MDS patients and is associated with worse prognosis with regard to transfusion frequency [53]. Of note, recent data demonstrate the increased propensity of highly purified MSCs from MDS patients to differentiate towards adipocytes [54], resembling bone marrow changes in obesity and aging [55]. Although these data for MDS are not unanimous [56], the increased adipogenic differentiation of MSCs and consequent support of leukemic progenitor cells are hallmarks of the related myeloid malignancy, AML [57].

However, metabolic processes in MDS MSCs have not been sufficiently investigated thus far, because in vitro research on MSC metabolism is biased by the high concentration of nutrients and growth factors in the culture medium. While low-passage MSCs maintain aerobic glycolysis as their primary metabolic pathway [58,59], their prolonged expansion leads to a switch towards OXPHOS [60,61]. To avoid such effects, modifications of culture conditions were proposed, such as induction of hypoxia [62,63] or usage of a HIF1α stabilizer (e.g., deferoxamine) [64], which prevents MSC senescence induced in cell culture by sustaining glycolysis and inhibiting OXPHOS.

Besides MSCs, several other cell types are hematopoietic niche components. As an example, endothelial cells line blood vessels and regulate the egress of mature blood cells from the bone marrow to the bloodstream. Dysfunction of endothelial cells and enhanced bone marrow vascularization are described in MDS [65]. The aberrant expression of HIF1α in endothelial cells represents a potential target for drugs such as lenalidomide, which has been found to have strong inhibitory effects on HIF1α expression besides its known anti-angiogenic effects [66].

### 3.1. Inflammation in the Bone Marrow (BM) Niche

BM niche cells contribute to the regulation of the hematopoietic response during high demand circumstances, such as inflammatory or infectious events [12]. Through paracrine secretion of cytokines and growth factors (such as G-CSF, IFNs, IL-6, IL-1, TGFβ, and TNFα), as well as direct interactions between niche cells and HSCs, the BM niche promotes the exit of HSCs from a quiescent state so that they can expand and differentiate into mature myeloid cells [12,67].

Similar to the metabolic plasticity of HSCs, immune cells (such as macrophages, neutrophils, and lymphocytes) also have different metabolic requirements depending on their function and differentiation. Equivalent to cancer cells, rapid expansion of immune cells requires a switch to glycolytic metabolism. Glycolytic reprogramming depends on activation of several enzymes, such as mTOR kinase, which is the primary regulator that promotes the entry of myeloid and lymphoid cells into a pro-inflammatory state [68,69,70]. In general, aerobic glycolysis is mainly used by cells with inflammatory activity and a high demand for proliferation, whereas cells with immunoregulatory or anti-inflammatory functions that are involved in cell repair primarily use FAO and the TCA cycle [71]. This metabolic adaptation according to phenotype is particularly evident in macrophages, which can be classified into two types: the highly glycolytic M1 type, which produce pro-inflammatory cytokines and ROS, and the anti-inflammatory M2 type, which primarily rely on FAO metabolism and OXPHOS, ensuring energy fuels during long-lasting repair processes [72].

Chronic inflammation of the bone marrow has been implicated in the development and progression of myeloid malignancies [73]. Especially in the early disease stages of MDS, an aberrant innate immune response might contribute to progression and maintenance of malignant clones. Indeed, inflammatory mediators, such as the danger-associated molecular pattern molecules S100A8 and S100A9, and pro-inflammatory cytokines, including IL-1β, IL-6, TNFα, and IFN-γ, are elevated in low-risk MDS [74]. The alarmin S100A9 protein serves as a key driver of the NLRP3 inflammasome complex; this mediates pyroptosis, a form of inflammatory cell death of HSCs [75] that promotes the MDS phenotype. S100A9 triggers the inflammasome by activating TLR4-mediated downstream signaling, including nuclear factor kappa B (NF-κB)-dependent transcription and secretion of pro-inflammatory cytokines. Furthermore, S100A9 modulates the metabolic features of HSCs through activation of NADPH oxidase and increased levels of ROS, which initiate cation influx [76]. Moreover, S100A8/S100A9 stimulates IL-1β production in adipose tissue macrophages, which, in turn, leads to stimulation of myelopoiesis in the bone marrow [77], thereby connecting hematopoiesis with adipose tissue inflammation in obesity.

Increasing evidence indicates a linkage among dysregulated metabolism, inflammation, and clonal hematopoiesis [78]. For instance, *Tet2*-deficiency in mouse HSPCs and *TET2*-mutant human HSPCs are associated with a clonal advantage in an inflammatory milieu [79]. The TET2 protein plays a role as an epigenetic regulator in myeloid differentiation and further inhibits expression of inflammatory factors, such as IL-6 [80]. Therefore, *TET2* mutations, which are increasingly detected in aging population [81], lead to the expansion of clonal hematopoiesis, particularly due to the development of resistance in the inflammatory micro-milieu produced by these clones. In *Tet2*-deficient mice, inflammatory macrophages express high levels of IL-1β, which leads to intensive monocyte recruitment and accelerates atherosclerosis development [82,83]. In humans, whole exome sequencing has demonstrated similar results and has shown that the presence of clonal hematopoiesis is associated with an elevated risk of cardiovascular disease [84].

Taken together, the combination of dysregulated metabolism and pro-inflammatory alterations generates a complex immunometabolic network in the bone marrow, which contributes to the pathogenesis of CHIP and MDS [10,18].

### 3.2. Iron Overload and Its Effect on Niche Metabolism in MDS

Iron overload (IO) is considered a hallmark of MDS (Figure 1) due to ineffective erythropoiesis and the need for chronic transfusions, which are a mainstay of supportive care [85]. It is difficult to distinguish age- or lifestyle-related metabolic changes in patients with MDS from those induced by iron overload; an additive effect may be assumed. However, a bidirectional effect is also conceivable. IO in the context of MDS may predispose an individual to the development of metabolic syndrome; the latter may contribute to the persistence of systemic inflammation and the propagation of malignant clones in the bone marrow niche.

At the molecular level, IO is associated with a reduction in the ATP/AMP ratio in mononuclear cells in MDS compared with age-matched healthy controls, which indicates an altered energy balance [86]. This reduction originates from a progressive inefficiency of OXPHOS, which is characterized by an increase in oxygen consumption and a decrease in ATP generation. Such uncoupled respiration leads not only to decreased energy production but also to increased production of ROS, which can then modify proteins, lipids, and DNA to promote genetic instability and further propagation of mutated clones [87].

Aberrant HIF1α signaling may couple the effect of IO on HSCs and niche cells in MDS. Overexpression of HIF1α in malignant myeloid cell lines attenuates the damage of erythroid progenitors induced by ROS due to the IO [88]. On the contrary, HIF1α elevation in MSCs leads to increased apoptosis in parallel with high ROS levels and elevation of MSC-secreted cytokines that are involved in the pathogenesis of MDS (e.g., IL-6, IL-8, TGFβ, and VEGF) [89]. Stabilization of HIF1α in MSCs prevents osteogenic differentiation and therefore alters the function of these cells and, in parallel, reduces mitochondrial biogenesis [34]. Further investigation of the role of HIF1α in the MDS micro-milieu is required, as various HIF inhibitors are currently being tested for different hematological and oncological disorders [90].

IO also contributes to cardiovascular pathology in MDS [91]. Over the last years, clonal hematopoiesis has been considered a distinct risk factor for the development of cardiovascular disorders, such as ischemic heart disease [84]. However, as an MDS-associated factor, IO can aggravate or even trigger atherosclerosis-like changes, including vascular impairment, inflammation, ROS production, and LDL oxidation, through the multifactorial pro-atherogenic action of non-transferrin-bound iron (NTBI) [92].

### 3.3. Relationship between the Microbiome and Metabolic Changes in the BM

The influence of the gut microbiota composition on hematopoiesis in the reactive state and hematologic malignancies has been proposed over the last 10 years [93]. An impairment in HSPC function and hematopoiesis leading to microbiome depletion has been demonstrated in mice treated with broad-spectrum antibiotics [94]. Importantly, in this mouse model, HSC cell cycle activity was suppressed and was accompanied by a maturation block in the final stages of granulocyte development, which resembles ineffective hematopoiesis in MDS [94].

A clear association between microbial evasion and pre-leukemic myeloproliferation has been shown in *Tet2*-deficient mice [95]. On one hand, these mice display increased intestinal permeability accompanied by the presence of *Lactobacillus* in the blood stream with consequent induction of systemic inflammation and IL-6 elevation; on the other hand, excessive myeloproliferation and extramedullary hematopoiesis resemble the clinical features of chronic myelomonocytic leukemia (CMML) [95,96]. Supporting this association, the use of germ-free mice or antibiotic treatment inhibited the growth of *Tet2*-deficient myeloid and lymphoid tumor cells in vivo and decreased inflammatory TNFα signaling in *Tet2* knockout mice [97]. Taken together, the shifts in the bacterial composition that occur in MDS, especially decreased microbial diversity, may conceivably lead to the suppression of normal hematopoiesis and therefore could contribute to the pathophysiology of cytopenia [94]. In addition, alterations in the microbiome may promote the emergence of malignant clones through inflammatory signaling, at least when certain molecular aberrations, such as *TET2* mutations, are present [95].

Although the gut microbiota of MDS patients has not yet been characterized, there is already evidence of its composition in the closely related myeloid neoplasm of AML. Decreased microbial diversity in AML patients predicts the development of infection and systemic inflammation during treatment [98,99,100], and conversely, the risk of developing secondary AML is significantly higher in individuals with prior infection and antibiotic use, which results in changes in microbial distribution [101]. The most abundant bacterial phyla in treatment-naïve AML patients without antibiotic exposure were Firmicutes (mostly Gram-positive bacteria including Clostridia, and Bacilli, which are able to regulate cholesterol homeostasis [102]), Bacteroidetes (produce succinic acid, acetic acid, and in some cases propionic acid, as major end products), Proteobacteria (including pathogenic Gram-negative bacteria, such as *Escherichia*, *Salmonella*, and *Vibrio*, among others), Verrucomicrobia, and Spirochaetes [103].

Taken together, the published data serve as a basis for considering microbiome changes as relevant players in the pathogenesis of MDS. Whether specific microorganisms or a general imbalance in gut microflora induce nonspecific inflammation to support malignant hematopoiesis remains to be determined. Therefore, host–microbial symbiosis represents an attractive research area that can improve our understanding of MDS pathogenesis and identify potential treatment options.

## 4. Summary and Outlook: Clinical and Therapeutic Aspects of Metabolic Changes in MDS Patients

Systemic metabolic changes that result in an elevated risk for cardiovascular diseases are epidemiologically and pathogenetically associated with a higher risk of developing clonal myeloid disorders, such as MDS [104]. Both cardiovascular and clonal hematological diseases are increasingly detected in the elderly population and are linked to age-related inflammatory processes.

At the cellular level, altered metabolic pathways in malignant cells lead to a survival advantage and expansion of malignant clones [78]. It is assumed that the surrounding bone marrow microenvironment adapts to new conditions based on its needs toward a pro-inflammatory milieu, which supports malignant clones [12]. The key regulators of associated metabolic pathways are also considered potential therapeutic targets in various malignant disorders [105], including myeloid malignancies [106].

A good example of a metabolically oriented MDS therapy is iron chelation, the primary effect of which was originally attributed to reduction in iron overload with consequent reduction in ROS and the pathological effects thereof on hematopoiesis. However, it has been shown that iron depletion exerts direct metabolic effects through improvement in insulin resistance in patients with hyperferritinemia [107]. According to retrospective analyses, iron chelation also delayed cardiac events in transfusion-dependent patients with MDS [108], whereas the randomized prospective TELESTO trial demonstrated that adverse events, including cardiac dysfunction, occurred approximately one year later in patients treated with iron chelation compared with placebo [91]. At the molecular level, iron chelators can directly improve the ATP/AMP ratio to partially restore mitochondrial function and reduce the malondialdehyde (MDA) level [86]. Therefore, manipulation of iron metabolic pathways represents a metabolism-modulating therapy in MDS patients.

Similarly, changes in metabolic activity are observed in the treatment of leukemia patients with the oral BCL-2 inhibitor venetoclax combined with demethylating agents. This combination, which is currently under investigation for MDS (e.g., clinical trials NCT04401748 and NCT04550442), eradicates leukemic stem cells by disrupting the TCA cycle [109] and decreasing amino acid uptake, which results in suppressed OXPHOS activity [110]. Other mutation-based therapies, specifically ivosidenib (IDH1 inhibitor) and enasidenib (IDH2 inhibitor), are the first FDA-approved drugs that target metabolism in AML patients, as they induce durable remissions by reducing the levels of the oncometabolite 2-HG [111,112], which leads to a differentiation blockade of AML cells [113].

Another intervention based on dietary caloric restriction has become increasingly recognized as a supportive therapeutic approach for various malignancies [114]. Dietary interventions lead to improvement in insulin sensitivity in nondiabetic individuals, whereas phosphatidylcholine and proline concentrations represent important predictors of response [115]. Hormonal changes induced by fasting might be of special interest in MDS [116,117], because changes in adiponectin, leptin, insulin, and IGF1 levels influence the apoptosis rate of HSCs in the bone marrow [116]. The potential of caloric restriction to reduce inflammation and its positive influence on the gut microbiome [118] suggest that this approach is worth exploring in MDS.

In addition to therapeutic approaches, understanding the MDS metabolome may also have prognostic implications. Recently, a panel including 15 metabolism-related genes was established and was determined to have better prognostic capability compared with the traditional international prognostic scoring system (IPSS) [119]. Whether this gene-based prognostic model will be applicable in clinical practice remains unclear.

Taken together, considering MDS a disease with specific metabolic changes supports both the identification of modes of action of already approved drugs and opens a wide field of potential novel targets for interference. Further clarification of the distinct role of the bone marrow microenvironment in the metabolic regulation of MDS clones will lead to novel treatment strategies for this malignancy.

## Data Availability

All data presented this study are available from the corresponding. Author, upon responsible request.
